# Cutaneous Squamous Cell Carcinoma (SCC) and the DNA Damage Response: pATM Expression Patterns in Pre-Malignant and Malignant Keratinocyte Skin Lesions

**DOI:** 10.1371/journal.pone.0021271

**Published:** 2011-07-01

**Authors:** Ferina Ismail, Mohamed Ikram, Karin Purdie, Catherine Harwood, Irene Leigh, Alan Storey

**Affiliations:** 1 Centre for Cutaneous Research, Blizard Institute of Cell and Molecular Science, Barts and The London School of Medicine and Dentistry, Queen Mary University of London, London, United Kingdom; 2 Molecular Oncology and Imaging, Institute of Cancer, Barts and The London School of Medicine and Dentistry, Queen Mary University of London, London, United Kingdom; 3 College of Medicine Dentistry and Nursing, University of Dundee, Dundee, United Kingdom; 4 Department of Molecular Oncology, Wetherall Insitute of Molecular Medicine, University of Oxford, John Radcliffe Hospital, Oxford, United Kingdom; University of Medicine and Dentistry of New Jersey, United States of America

## Abstract

Recent evidence suggests that an initial barrier to the emergence of tumours is a DNA damage response that evokes a counter-response which arrests the growth of, or eliminates, damaged cells. Early precursor lesions express markers of an activated DNA damage response in several types of tumour, with a diminishing response in more advanced cancers. An important marker of DNA damage is ATM which becomes phosphorylated (pATM) upon activation. We have investigated pATM expression patterns in cultured keratinocytes, skin explants and a spectrum of pre-malignant to malignant keratinocyte skin lesions by immunohistochemistry. We found that pATM was mainly localised to the Golgi apparatus, which contrasts with its nuclear localisation in other tissues. Upon UV irradiation there is transient formation of pATM in nuclear foci, consistent with recruitment to the sites of DNA damage. By immunohistochemistry we show pATM expression in precancerous keratinocyte lesions is greater and predominantly nuclear when compared to the invasive lesions where pATM is weaker and predominantly cytoplasmic. Our results are consistent with the hypothesis that the DNA damage response acts as a barrier to cutaneous tumour formation, but also suggests that ATM expression in skin is different compared to other tissues. This may be a consequence of the constant exposure of skin to UVR, and has implications for skin carcinogenesis.

## Introduction

The integrity of genomic DNA is constantly under threat from cytotoxic agents. DNA damage checkpoints ensure genomic stability both by delaying cell cycle progression and by activating repair processes, pathways essential for prevention of tumour development. If the cell is damaged beyond repair then these checkpoints can also participate in apoptosis to eliminate the damaged cells. Central to the DNA damage response is the protein kinase ATM, a member of the phosphatidyl inositol 3-kinase-like-kinase (PIKK) family. ATM is required for transduction of the DNA damage signal to downstream protein kinases essential for the execution of cell cycle checkpoint arrest [1]. In an unstimulated state, ATM is thought to exist as a homodimer in which the kinase domain of one subunit faces the autophosphorylation site of another [2]. Upon activation, ATM undergoes a conformational change that stimulates the kinase to phosphorylate Ser1981 by intermolecular autophosphorylation (pATM), resulting in dissociation of the homodimer [2]. Several downstream targets of pATM have been identified including p53, Mdm2, E2F1, Chk2, Nbs1, histone H2AX, c-Abl and BRCA1. These, in turn, are responsible for multiple downstream effects including activation of the G1/S, S and G2/M checkpoints and DNA repair activation. Activation of ATM and the downstream cell cycle checkpoint kinases Chk1 and Chk2 constitute an important mechanism for eliminating damaged cells early in precancerous lesions [3]. DNA damage checkpoints might become activated in the early stages of human tumorigenesis, leading to cell cycle blockade or apoptosis, thereby hindering tumour progression. Recent evidence suggests that an initial barrier to the emergence of tumour cells is a DNA damage response that evokes a counter-response responsible for eliminating damaged and potentially dangerous cells [4], [5]. In clinical specimens from different stages of human tumours (including urinary bladder, breast, lung and colon), the early precursor lesions (but not normal tissues), express markers of an activated DNA damage response. These included phosphorylated kinases ATM and Chk2, and phosphorylated histone H2AX and p53.

As yet, no similar studies have been reported in cutaneous squamous cell carcinoma (SCC), although Gorgoulis et al. examined the DNA damage response in dysplastic naevi and melanomas, as well as adjacent normal skin [3]. Non melanoma skin cancers (NMSC), including cutaneous SCC, are the most common human malignancies and cutaneous SCC provides an ideal model in which to study the DNA damage response since there exists a spectrum of stages in its development, from premalignant actinic keratosis through to carcinoma *in situ* (Bowen's disease), and invasive SCC. UVR is the principle carcinogen implicated and there is overwhelming epidemiological evidence to support an association between UV exposure and SCC reviewed by Armstrong and Kricker, 2001 [6]. Solar radiation, in particular the UVB component of the spectrum, is known to induce mutations in genomic [7] and mitochondrial DNA [8], which makes it the most important aetiological agent in the development of NMSC [9].

UV-induced DNA damage is characterised by two main types of lesions: cyclobutane pyrimidine dimers (CPDs) and (6–4) photoproducts. Translesional DNA synthesis by pol 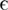 can lead to mis-incorporation of nucleotides, resulting in genomic mutations which may then lead to cancer [10]. The importance of effective DNA repair is highlighted by the photosensitive inherited disorder xeroderma pigmentosum (XP) in which patients have a defect in nucleotide excision repair (NER)-associated genes and are sensitive to UV exposure, with an increased susceptibility to NMSC [11]. Recent studies in mice transgenic for either a (6–4) photoproduct or CPD photolyase gene, whose expression was targeted to basal keratinocytes, revealed the importance of the removal of CPDs in preventing NMSC [12]. Furthermore, transcriptome analysis showed that the most prominent pathway induced by CPDs was that associated with double strand break (DSB) repair. These results imply that the conversion of unrepaired CPDs into dsDNA breaks during DNA replication constitutes the principal source of UV-mediated cytotoxicity [13]. ATM is believed to be predominantly activated by DSBs, lesions that are highly genotoxic. Given that UVR primarily induces DSBs [13], the aims of this study were to investigate the expression of ATM and activated ATM (pATM) in both normal skin as well as in the spectrum of premalignant actinic keratosis (AK) to carcinoma *in-situ* (CIS) to invasive SCC lesions, to see whether the model proposed by Bartkova et al. could be extended to include cutaneous SCCs. It was also hypothesised that, as skin is constantly exposed to UVB, there may be differences in cutaneous / keratinocyte ATM function.

## Results

### pATM is localised to the Golgi apparatus in normal human primary keratinocytes (NHPK) following UVB irradiation

**Figure 1:**
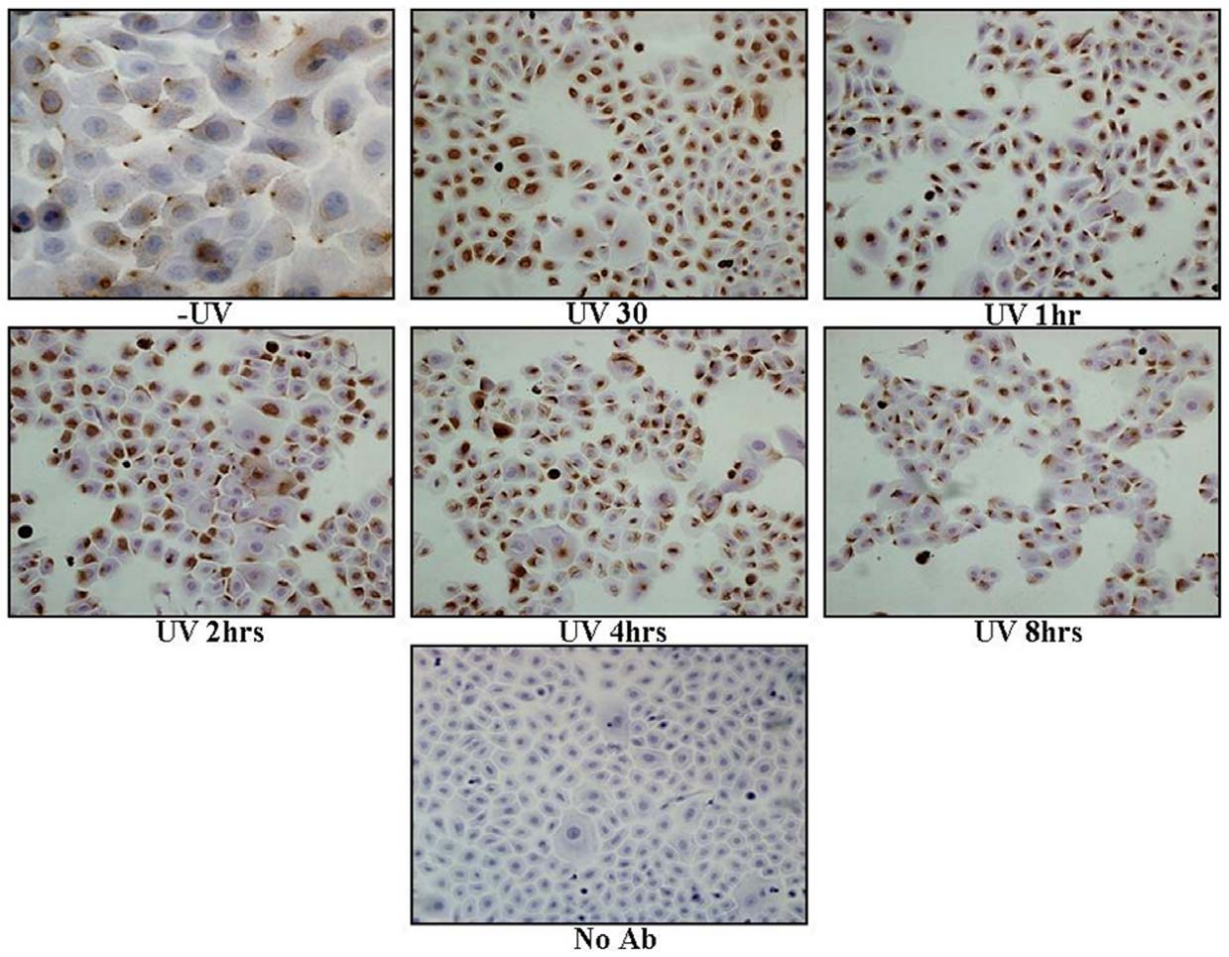
pATM staining of Normal Human Primary Keratinocytes +/− UVB. Cells were UVB irradiated (10 mJ/cm^2^) and fixed at various times post-UV with 4% PFA. Un-irradiated cells show cytoplasmic expression of pATM with more nuclear staining observed 30–60 minutes post-UV. 2 hours following UV irradiation pATM again assumes perinuclear and cytoplasmic expression.

**Figure 2:**
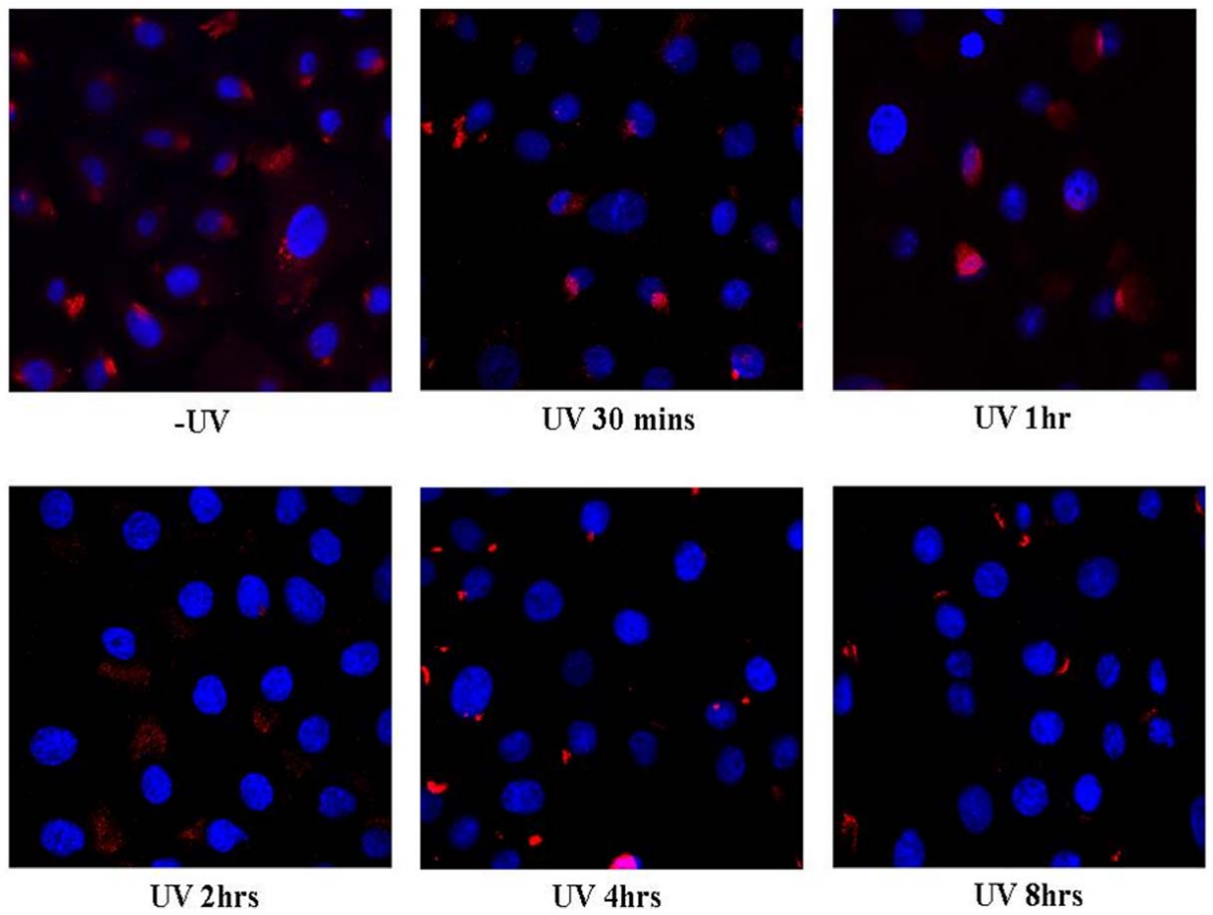
Fluorescent pATM staining of NHPK +/− UVB. Same cells as in Fig 1 fluorescently labelled using DAPI (blue) to stain the nucleus. pATM was fluorescently labelled with Alexa Fluor 568 (red) and cells visualised using confocal microscopy. Findings mirror those observed in Fig 1 - a very specific pattern of cytoplasmic staining is seen; at 60 minutes following UVB there is more nuclear expression of pATM which is short-lived, returning back to the cytoplasm after 2 hours.

**Figure 3:**
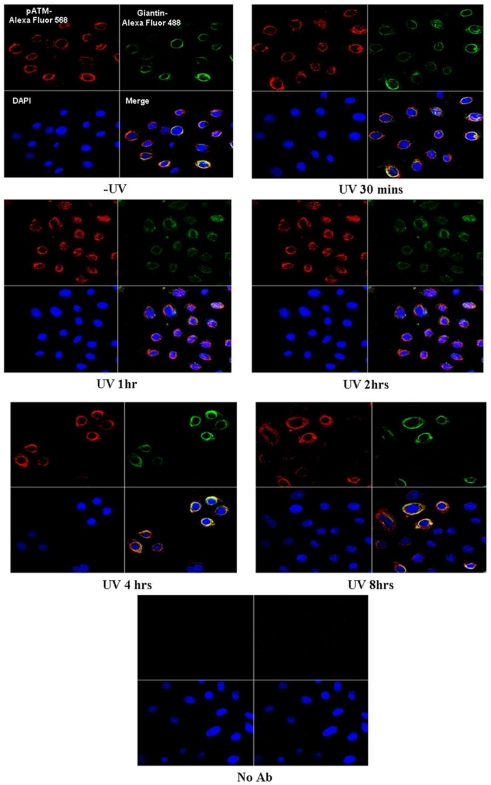
Confocal microscope image of fluorescently labelled NHPK +/− UVB with pATM and Golgi specific marker. Fluorescently labelled NHPK +/− UVB (10 mJ/cm^2^) with pATM-conjugated Alexa Fluor 568 (red), Golgi-specific antibody (Giantin) conjugated Alexa Fluor 488 (green), and nuclear DAPI staining (blue). There is co-localisation of pATM to the Golgi apparatus. 30 minutes to 2 hours post-UV there is transient nuclear localisation of pATM.

Normal human primary keratinocytes (NHPK) (derived from skin obtained from a non UV exposed site in a 30-year-old Caucasian woman), were irradiated with UVB and fixed at various time points post-treatment. Cells were stained for pATM both fluorescently and non-fluorescently (Figures 1 and 2). pATM in untreated NHPK was present at specific peri-nuclear foci. At 30-60 minutes post-UVB irradiation a significant proportion of pATM is expressed in the nucleus. However, 2 hours post-UV no nuclear expression of pATM was observed but instead pATM was once again found in its ‘resting position’ in the peri-nuclear and cytoplasmic regions of the cell. The specific peri-nuclear pattern of staining of pATM was postulated to represent localisation to the Golgi apparatus. To test this, NHPK cells were fluorescently labelled with both a Golgi specific marker (Giantin) and pATM (Fig 3). This double labelling demonstrates co-localisation of pATM to the Golgi apparatus. In untreated NHPK, pATM localised predominantly to the Golgi apparatus, although between 30 minutes to 2 hours post-UV irradiation, pATM was additionally visualised in the nucleus (consistent with the findings in Figs 1 and 2). Thus following UV, transient nuclear re-localisation of pATM can be seen after 30 minutes which then returns to the Golgi after 2 hours. The specificity of the pATM antibody used in this study is demonstrated in Figure S1 where a Western blot of the nuclear fraction of undamaged and UV irradiated normal human primary keratinocytes is labeled with both pATM and ATM antibodies. Following UV there is upregulation of nuclear pATM 1 hour post-UV with levels returning to baseline thereafter, and with no significant change in ATM expression.

### pATM expression in acute vs. chronically UV exposed normal skin differs, with greater nuclear expression seen in the latter

**Figure 4:**
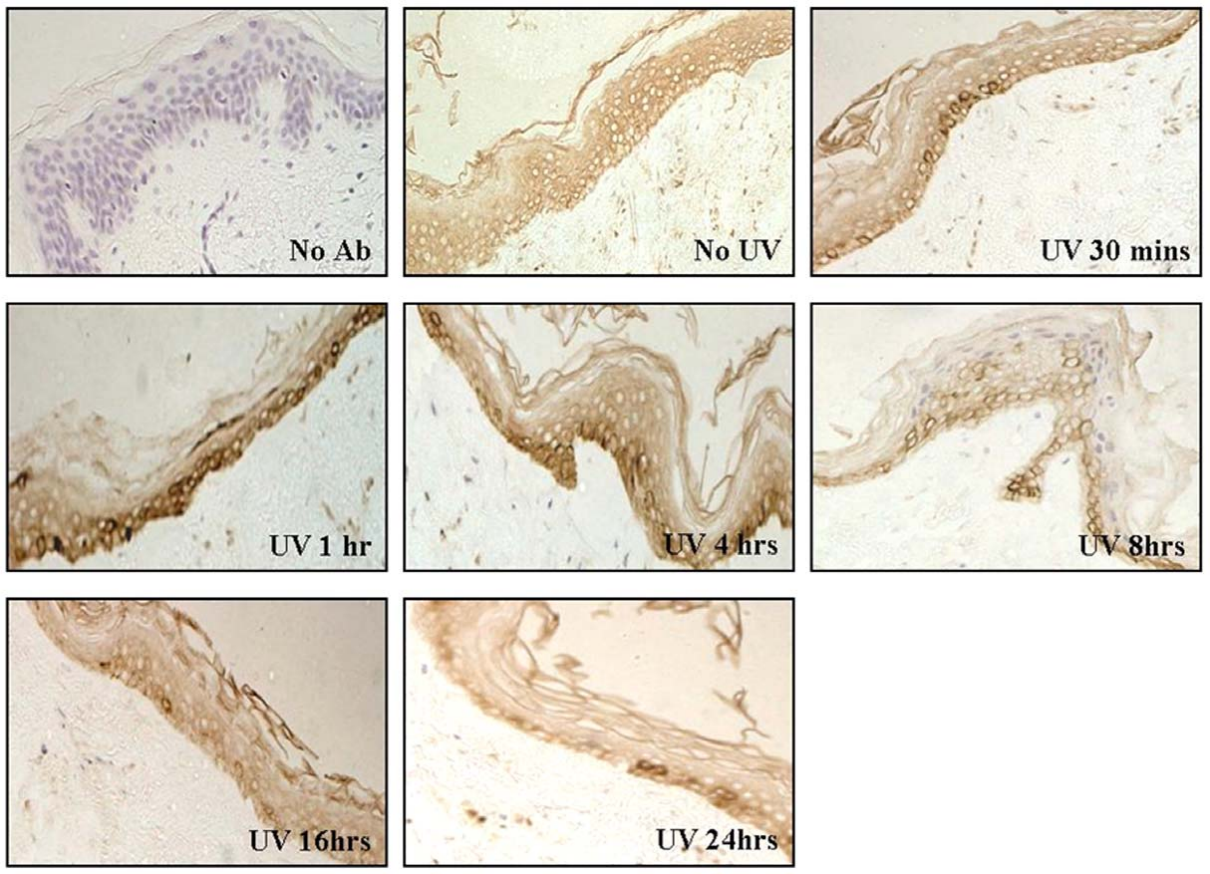
pATM expression in normal human skin +/− UVB. Paraffin embedded normal skin (taken from a non-UV exposed site) was UV irradiated (10 mJ/cm^2^) and fixed with 4% PFA at various times post-UV, then stained with pATM antibody at various time points post-UV. pATM expression can be seen in untreated skin with upregulation 30–60 minutes following UVB irradiation. pATM expression is predominantly cytoplasmic and peri-nuclear.

pATM expression was examined in untreated and UVB irradiated skin sections from explant organ culture. Normal skin was obtained from both a non UV-exposed and UV-exposed site in age- and sex-matched Caucasian subjects. Fig 4 shows immunohistochemistry of normal non-UV exposed skin irradiated with UVB. pATM expression can be seen in untreated skin which most likely represents either background staining or a low level of ‘real’ staining. There is upregulation of pATM 30–60 minutes post-UVB, in keeping with the well-documented rapid onset of pATM activation in response to a damaging stimulus. The upregulation of pATM was characterised by the visibly heavier expression in some cells, but not all; this appeared to be more prominent in the basal layers of the epidermis, presumably related to the greater proliferative potential of basal keratinocytes. Markedly increased expression at 1 and 4 hours post-UV with a reduction at 8 hours was observed. By 16 and 24 hours post-UV, only individual cells showed more intense staining compared to background levels. pATM expression was predominantly localised to the cytoplasm, particularly around the peri-nuclear region, with relatively sparse staining in the nucleus, consistent with observations in NHPK.

The UV-induced pATM signal demonstrated by immunohistochemistry is depleted by silencing of ATM expression. ATM in human keratinocytes (PM1) was silenced using SiRNA (Dharmacon) (Figure S2). Following UV irradiation there is upregulation of pATM in the scrambled cell line with significant nuclear staining after 1–2 hours. In contrast there is very little pATM seen in both undamaged and UVB irradiated ATM silenced cells (Figure S3).

**Figure 5:**
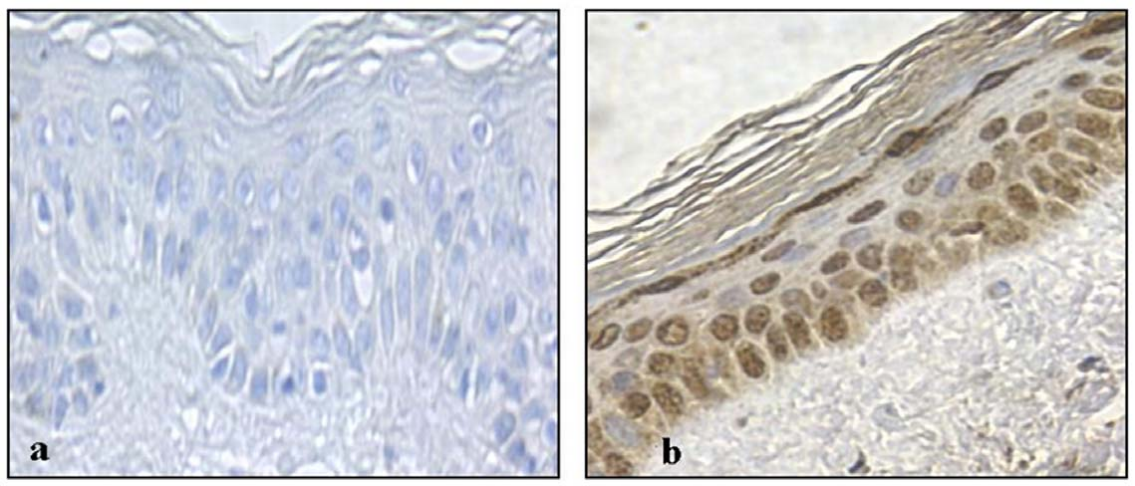
pATM expression in normal UV exposed skin (face). a) Negative control – no primary pATM Ab; b) pATM staining: predominantly nuclear staining observed.

Sections of biopsies from UV-exposed normal skin stained for pATM showed predominantly nuclear expression (Fig 5). This suggested that chronic rather than acute UV exposure is required as a stimulus for nuclear pATM expression even though UV exposure in the short term leads to a transient increase in pATM levels.

### ATM expression in pre-invasive keratinocyte intraepithelial neoplasia differs from that in invasive lesions

Investigation of pATM expression was extended to human skin malignancies by examining representative samples from each stage in SCC development.

#### (i) pATM in cell lines representing pre-invasive (PM1) and invasive (Met1) SCC show greater nuclear expression in the pre-invasive tissue

**Figure 6:**
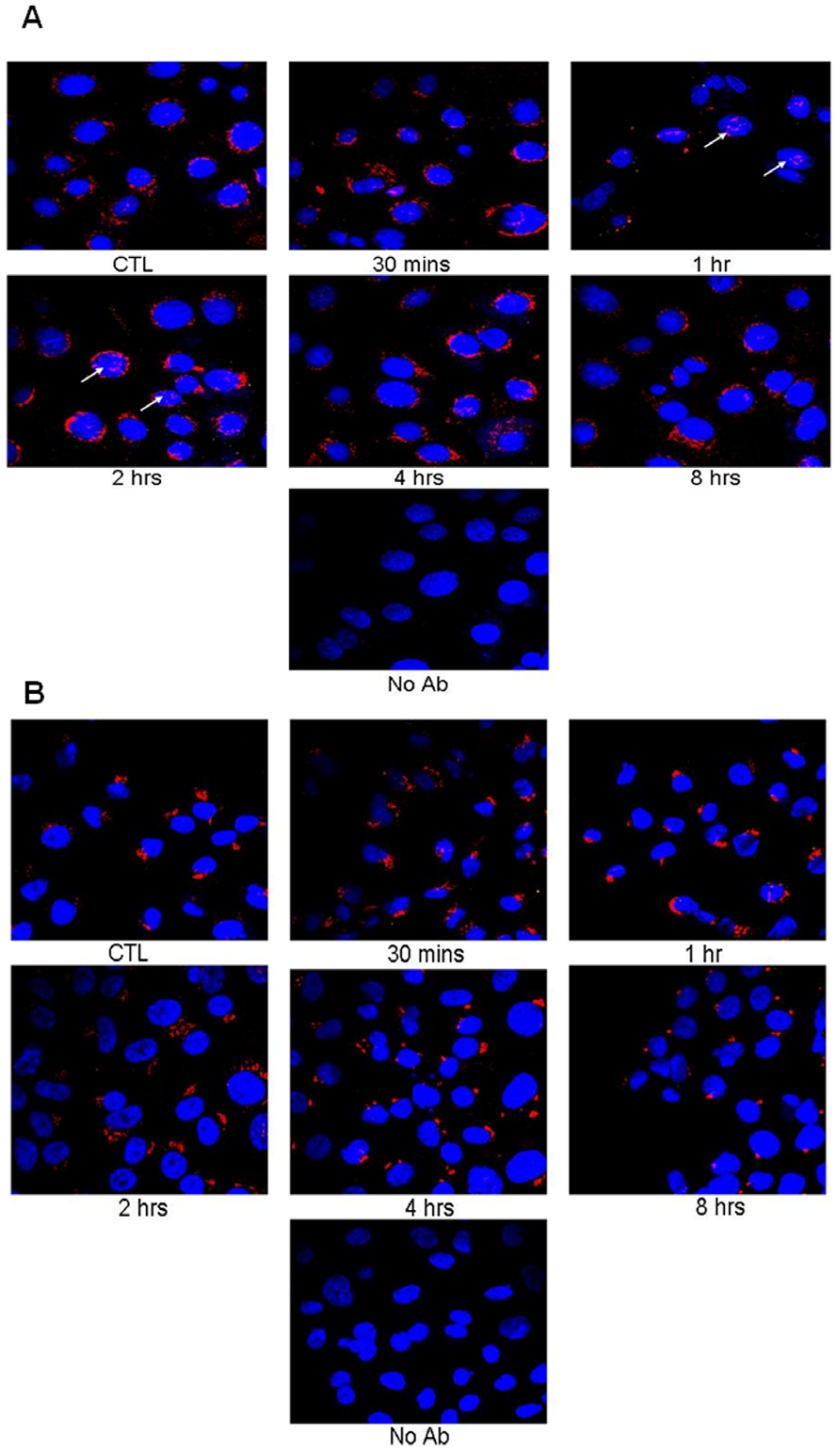
Fluorescent pATM staining of PM1 (A) and Met1 (B) cells +/− UV. PM1 and Met1 cells were UVB irradiated (10 mJ/cm^2^) and fixed in 4% PFA at various times post-UV. Cells were then labelled with pATM conjugated with Alexa Fluor 568 (red) and counterstained with DAPI (blue). (A) Note nuclear foci of pATM at 1 hour and 2 hours post-UV (white arrows) in PM1 cells. No nuclear expression of pATM observed in Met1 cells.

The cell lines PM1 and Met1 are derived from the same patient, a 55 year old Caucasian renal transplant recipient. The PM1 cell line was cultured from a dysplastic region on the forehead and Met1 from an SCC on the dorsum of hand which had metastasized; metastases were pathologically and genetically confirmed to have come from the primary SCC on the hand [14]. Cells were UVB irradiated and fluorescently stained with pATM. It can be seen in Fig 6 that both PM1 and Met1 cells have patterns of pATM expression similar to those seen in the NHPK, i.e. focal cytoplasmic and peri-nuclear staining consistent with Golgi localisation. No nuclear expression of pATM is seen in the Met1 cell line though there does appear be some, albeit very low levels, of pATM in the nucleus in the PM1 cell line, particularly 2 hours post-UVB treatment. Since the PM1 cells represent an early stage of tumorigenesis, this finding would be in keeping with the proposal that there is an upregulation of DNA damage response proteins in the nucleus in early carcinogenesis.

#### (ii) pATM in human skin samples representing sequential keratinocyte neoplasia show greater, and predominantly nuclear expression, in early lesions

In order to consolidate these preliminary findings a series of AKs, CIS and SCC lesions were analysed for pATM expression. Samples from each pathologic category were identified through the histopathology database and the histological diagnosis confirmed by re-examination of the H&E sections by two independent observers (RC and CH). AK sections were graded into three categories: AK I, II and III, based on the degree of atypical keratinocytes in the epidermis [15], [16]. SCCs were also classified into well, moderately and poorly differentiated tumours. Poorly differentiated tumours have a worse prognosis with more than double the local recurrence rate and triple the metastatic rate of well differentiated SCC [17].

#### Methods of Analysis

**Figure 7:**
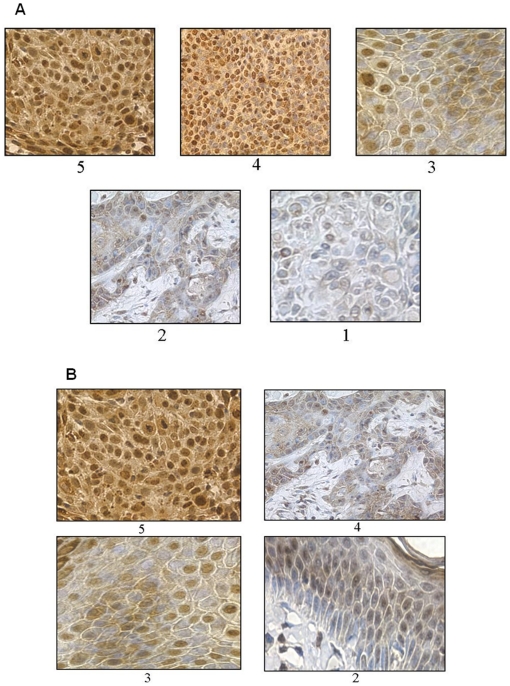
pATM expression in lesional tissue – examples of scoring for nuclear (A) and cytoplasmic (B) staining.

The extent of pATM expression was expressed as a percentage of positive cells with nuclear and cytoplasmic expression recorded separately. This was categorised as grade 1, >0–5%; grade 2, >5–25%; grade 3, >25–50%; grade 4, >50–75%; grade 5, >75–100%. Lesions were scored independently by two observers (FI and CH) and a consensus decision was reached in the case of any discrepancies (Table 1, Fig 7). We compared nuclear and cytoplasmic pATM expression using a paired t-test for matched data. pATM differences between cytoplasm and nucleus were compared across two groups using the rank sum test. pATM differences between cytoplasm and nucleus were compared across three ordered groups using the nonparametric test for trend across ordered groups developed by Cuzick. In addition, pATM was reclassified as a binary variable (≤50% vs. >50%) (Table 2). Comparisons between nuclear and cytoplasmic expression were made by comparing the discordant pairs (McNemar's chi-squared).

**Table 1:** pATM protein expression by immunohistochemistry – results for individual AK, CIS and SCC lesions.

		SCC				CIS	AK		
Grade of pATM protein expression		Well	Moderate	Poor	Total	Total	Grade I	Grade II	Total
>0–5%	**Nuclear**	0 (0.0%)	0 (0.0%)	1 (14.3%)	**1 (3.6%)**	**0 (0.0%)**	0 (0.0%)	0 (0.0%)	**0 (0.0%)**
	**Cytoplasm**	0 (0.0%)	0 (0.0%)	0 (0.0%)	**0 (0.0%)**	**0 (0.0%)**	0 (0.0%)	0 (0.0%)	**0 (0.0%)**
>5–25%	**Nuclear**	2 (28.6%)	4 (28.6%)	2 (28.6%)	**8 (28.6%)**	**0 (0.0%)**	0 (0.0%)	0 (0.0%)	**0 (0.0%)**
	**Cytoplasm**	2 (28.6%)	0 (0.0%)	2 (28.6%)	**4 (14.3%)**	**0 (0.0%)**	0 (0.0%)	0 (0.0%)	**0 (0.0%)**
>25–50%	**Nuclear**	3 (42.9%)	4 (28.6%)	3 (42.9%)	**10 (35.7%)**	**2 (7.1%)**	0 (0.0%)	0 (0.0%)	**0 (0.0%)**
	**Cytoplasm**	2 (28.6%)	0 (0.0%)	1 (14.3%)	**3 (10.7%)**	**1 (3.6%)**	1 (9.1%)	1 (20.0%)	**2 (12.5%)**
>50–75%	**Nuclear**	2 (58.6%)	5 (35.7%)	1 (14.3%)	**8 (28.6%)**	**7 (25.0%)**	1 (9.1%)	1 (20.0%)	**2 (12.5%)**
	**Cytoplasm**	1 (14.3%)	9 (64.3%)	2 (28.6%)	**12 (42.9%)**	**5 (17.9%)**	2 (18.2%)	0 (0.0%)	**2 (12.5%)**
>75–100%	**Nuclear**	0 (0.0%)	1 (7.1%)	0 (0.0%)	**1 (3.6%)**	**19 (67.9%)**	10 (90.9%)	4 (80.0%)	**14 (87.5%)**
	**Cytoplasm**	2 (28.6%)	5 (35.7%)	2 (28.6%)	**9 (32.1%)**	**22 (78.6%)**	8 (72.7%)	4 (80.0%)	**12 (75.0%)**
**Total**		7 (100%)	14 (100%)	7 (100%)	**28 (100%)**	**28 (100%)**	11 (100%)	5 (100%)	**16 (100%)**

**Table 2:** Summary of Nuclear vs. Cytoplasmic staining in AKs, CIS and SCC.

	Nuclear	Cytoplasm
AK	16/16	14/16
CIS	26/28	27/28
SCC	9/28	21/28

Figures only include grade of protein expression of >50%.

#### Comparison of Nuclear pATM

The majority of SCC lesions had nuclear pATM expression of between 5 and 75% with only 1/28 (3.6%) with >75% and 1/28 (3.6%) with ≤5%. There was no significant difference in pATM nuclear expression according to SCC differentiation status (p = 0.44). Similarly for AK lesions, there was no significant difference in pATM nuclear expression between AK grades (p = 0.44). The median pATM nuclear levels decreased from AK (75–100%) to CIS (75–100%) to SCC (25–50%) (p<0.001).

#### Comparison of cytoplasmic pATM

There was no significant difference in pATM cytoplasmic expression according to SCC differentiation status (p = 0.80). Similarly for AK lesions, there was no significant difference in pATM nuclear expression between grades (p = 0.88). The median pATM cytoplasmic expression decreased from AK (75–100%) to CIS (75–100%) to SCC (50–75%) (p = 0.002).

#### Difference between nuclear and cytoplasmic pATM

Within SCC lesions, pATM levels were higher in the cytoplasm then the nucleus in 16/28 (57.1%) lesions; equivalent in 11/28 (39.3%) lesions and lower in 1/28 (3.6%) lesions (p-value = 0.0001).

Among well differentiated SCC lesions, pATM levels were higher in the cytoplasm then the nucleus in 3/7 lesions; equal in 3/7 lesions and lower in 1/7 lesions (p-value = 0.45). 2/7 lesions had cytoplasmic pATM >50% but nuclear ≤50%, whereas 1/7 lesions had nuclear pATM >50% but cytoplasmic ≤50% (p = 1.0). Among moderately differentiated SCCs, pATM levels were higher in the cytoplasm then the nucleus in 9/14 lesions; equal in 5/14 lesions and was not lower in any lesions (p-value = 0.0006). 8/14 lesions had cytoplasmic pATM >50% but nuclear ≤50%, whereas no lesions had nuclear pATM >50% and cytoplasmic ≤50% (p = 0.0078). Among poorly differentiated SCCs, pATM levels were higher in the cytoplasm then the nucleus in 4/7 lesions; equal in 3/7 lesions and was not lower in any lesions (p-value = 0.038). 3/7 lesions had cytoplasmic pATM >50% but nuclear ≤50%, whereas no lesions had nuclear pATM >50% but cytoplasmic ≤50% (p = 0.25). In summary, the difference in pATM between cytoplasm and nucleus was not significantly different between differentiations in the SCC lesions (p = 0.45).

Among CIS lesions, pATM levels were higher in the cytoplasm then the nucleus in 4/28 (14.3%) lesions; equal in 23/28 (82.1%) lesions and lower in 1/28 (3.6%) lesions (p-value = 0.16). 2/28 (7.1%) lesions had cytoplasmic pATM >50% but nuclear ≤50%, whereas 1/28 (3.6%) lesions had nuclear pATM >50% but cytoplasmic ≤50% (p = 1.0).

Among AK lesions, pATM levels were higher in the cytoplasm then the nucleus in 1/16 (6.3%) lesions; equal in 11/16 (68.8%) lesions and lower in 4/16 (25.0%) lesions (p-value = 0.16). No lesions had cytoplasmic pATM >50% but nuclear ≤50%, whereas 2/16 (12.5%) lesions had nuclear pATM >50% but cytoplasmic ≤50% (p = 0.5). There were no significant differences according to grade of AK.

**Figure 8:**
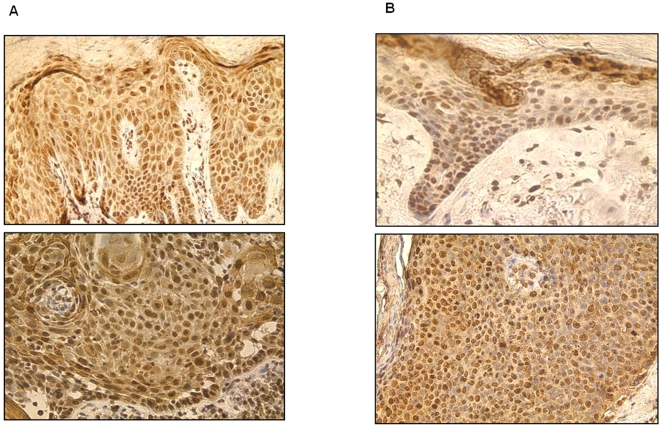
pATM expression in AK. A) Scored as nuclear 5 and cytoplasm 5; B) Scored as nuclear 4 and cytoplasm 3.

**Figure 9:**
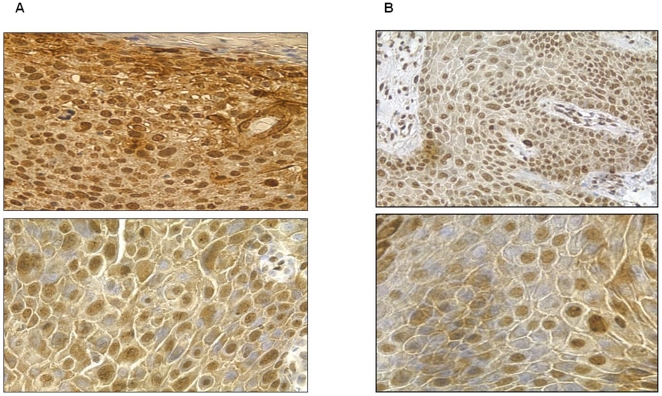
pATM expression in CIS. A) Scored as nuclear 5 and cytoplasm 5; B) Scored as nuclear 4 and cytoplasm 4.

**Figure 10:**
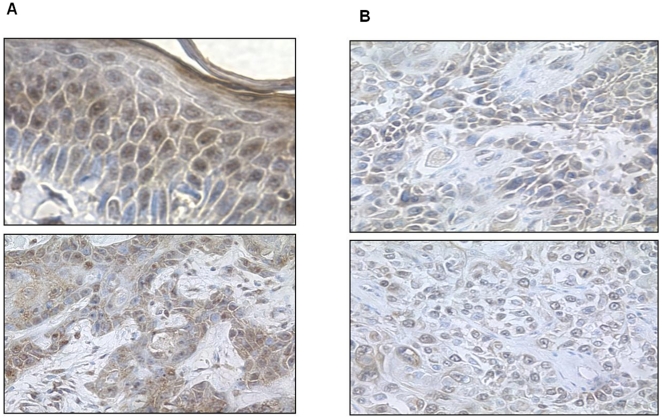
pATM expression in SCCs. A) Scored as nuclear 3, cytoplasm 4; B) Scored as nuclear 2, cytoplasm 4.

**Figure 11:**
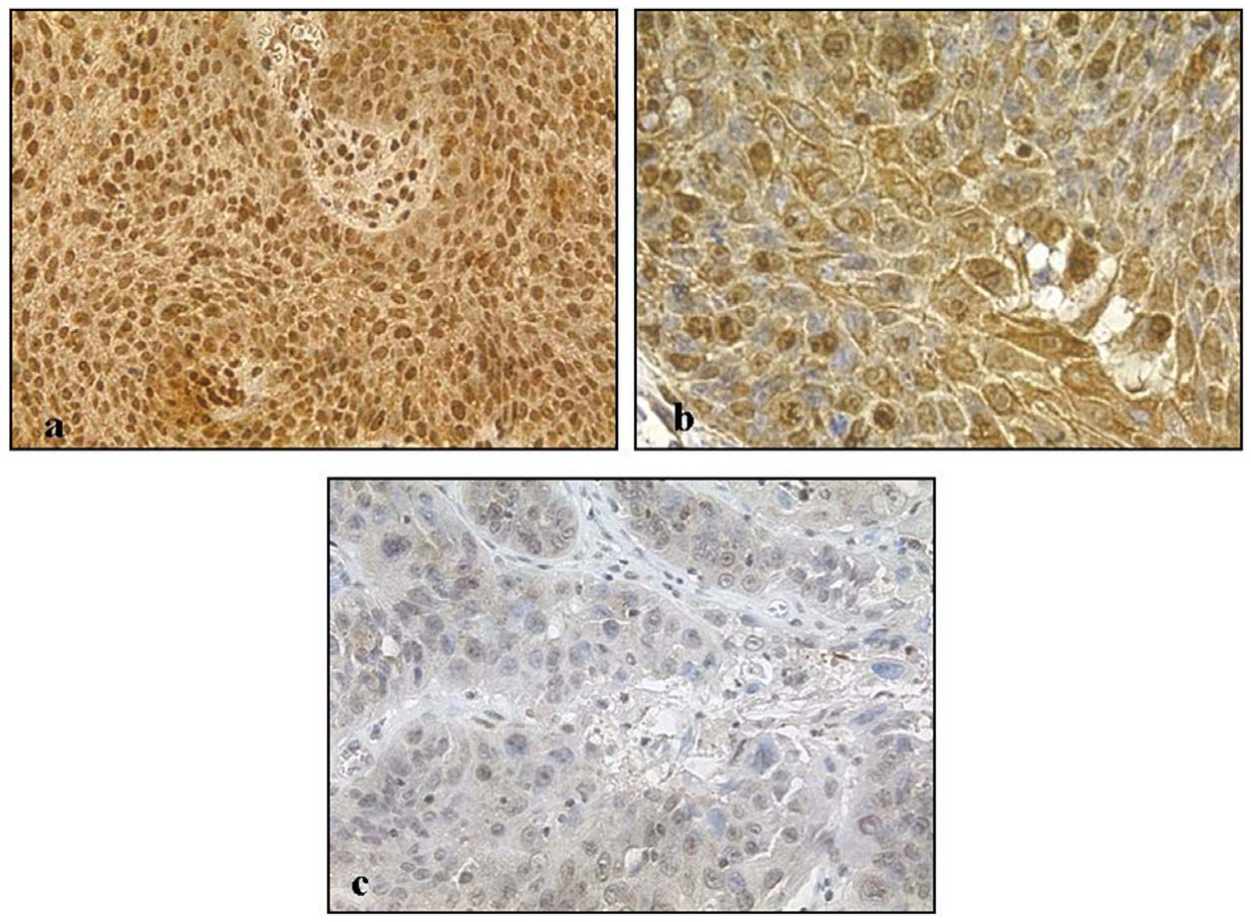
Overall pattern of pATM expression observed in spectrum of AK / CIS / SCC. This demonstrates greater proportion and increased intensity of nuclear staining of earlier lesions i.e. AK (a) and CIS (b) compared to less extensive and weaker nuclear staining in more advanced SCC (c).

Figs 8, 9, 10 show representative pATM stained sections of AKs, CIS and SCC lesions respectively, with Fig 11 demonstrating more clearly the overall differences in expression pattern that exist between the histological spectrum of disease. AKs show predominantly heavy nuclear expression of pATM which becomes progressively less nuclear and more cytoplasmic as the lesion evolves to more advanced SCC. It is also notable that, where present, nuclear expression of pATM was detected in histologically normal perilesional skin. This accords well with the observation that pATM is also predominantly nuclear in normal skin from chronically UV exposed sites, similar to the expression pattern seen here in precancerous AKs and CIS.

## Discussion

This is the first study to date to investigate the localisation and behaviour of pATM in skin in an attempt to address the proposal that an activated DNA damage response is an initial barrier to the emergence of cutaneous SCC, as has been postulated for other cancers [4], [5]. Key findings from these studies show that in clinical specimens from different stages of various other tumours that the early precursor lesions, but not normal tissues, commonly express markers of an activated DNA damage response. Genetic analyses indicated that early in tumourigenesis (before genomic instability and malignant conversion), human cells activate an ATR/ATM-regulated DNA damage response network that delays or prevents cancer. Data presented in this study provide evidence that the DNA damage response is active in all pre-invasive lesions (AK and CIS) but that this mechanism is overridden at a later point in the evolution of cutaneous SCC.

In earlier studies, the pATM expression pattern reported in precancerous lesions from tissues other than skin was predominantly nuclear, with expression levels diminishing in more advanced lesions [4]. In skin we have found that even in the presence of nuclear expression of pATM, precancerous AKs can theoretically still progress to CIS given that a similar level of nuclear pATM expression exists in both stages of skin cancer development. A transition appears to occur between CIS and SCC, with a significant loss of nuclear pATM expression seen in the latter. Several preliminary conclusions may be drawn from these findings. Firstly, it is apparent that the DNA damage response in skin behaves differently to other tissues. The predominance of pATM in the cytoplasm and Golgi apparatus suggests alternative roles for the protein other than as a sensor of DNA damage. Although the presence of ATM in cytoplasmic compartments has been previously documented in other tissues [18], [19], it has not been reported to occur in skin and not at such high levels in comparison with nuclear expression. This difference in DNA damage response is perhaps not surprising given the constant exposure of skin to UVR. Further evidence indicating the unique nature of skin with regard to its environmental susceptibility is also possibly reflected in the differences in distribution and levels of pATM in acute versus chronically UV exposed skin (i.e. cytoplasmic and nuclear respectively).

### Expression of pATM in normal skin is both cytoplasmic and nuclear

In proliferating cells ATM is predominantly nuclear, in keeping with a role in DNA-damage recognition and cell cycle control. A proportion of ATM is extranuclear even in proliferating cells and is predominantly cytoplasmic in post-mitotic cells [18], [20]–[23]. The extranuclear ATM in proliferating cells is primarily, if not entirely, present in vesicles including peroxisomes and endosomes [19], [24]. With regards to active, phosphorylated ATM (pATM), nearly all studies report its presence in the nucleus in several tissue types including breast, colon and bladder [4], in keeping with its role in sensing DNA damage. However, Wu et al, using subcellular fractionation studies, demonstrated that a small proportion of activated ATM was exported from the nucleus in a NEMO-dependent (NF-κB essential modulator) manner in HEK293 cells [25]. All reports of the cytoplasmic localisation of ATM are from studies involving the use of tissues and cell lines of non-cutaneous origin, and the subcellular localisation of ATM has not been previously reported in skin. A significant proportion of the active,phosphorylated form, pATM, is also extranuclear, as shown in this study, a finding not previously reported in skin and rarely addressed in non-cutaneous tissue.

### Cytoplasmic pATM is localised to the Golgi apparatus and is transiently expressed in the nucleus upon UV irradiation

In NHPK monolayer cells fluorescently labeled with both pATM and a Golgi specific marker, pATM co-localises to the Golgi apparatus (Fig 3). Upon UV irradiation there is transient expression of pATM in nuclear foci consistent with its recruitment to the sites of DNA damage. Previous studies of pATM expression have reported its presence in the nucleus in several tissue types including breast, colon and bladder [4], in keeping with its role in sensing DNA damage.

### Possible role of cytoplasmic pATM in skin

The results presented here suggest that the DNA damage response in skin may be different compared to other tissues and that pATM could have other functions. This may be a result of the skin's constant exposure to UV irradiation, and has implications for skin carcinogenesis. It is likely that nuclear pATM is involved in the DNA damage response and that cytoplasmic pATM may have other roles. A number of additional functions other than its role in DNA damage have previously been proposed for ATM though no clear understanding of these functions has been fully established. ATM associates with β-adaptin in cytoplasmic vesicles (in human lymphoblasts) which suggests a role in intracellular transport mechanisms [24]. Zhang et al. show that ATM is an interacting partner of CKIP-1 (casein kinase-2 interacting protein), a protein implicated in muscle differentiation, regulation of cell morphology and actin cytoskeleton. Although CKIP-1 is localised both in the nucleus and the plasma membrane, only plasma membrane-localised CKIP could form a complex with ATM and recruited nuclear ATM protein to the plasma membrane, including the phosphorylated form [26].

Molecular linkage between ATM and NF-κB signalling has also been shown [25], with the NF-κB family of transcription factors regulating genes involved in development, immune responses, cell migration and apoptosis [27]. The observation by Yang and Kastan that insulin enhances the activity of ATM kinase supports a more general signalling function for ATM [28]. They provide evidence that the kinase activity of ATM is also activated by insulin through a non-DNA damage signalling pathway. It should be noted that none of these studies involve the use of cutaneous tissue. Finally, the role of ATM in vesicular structures has been postulated to represent a specialised antioxidant system responsible for detoxifying reactive oxygen intermediates although this is speculative [23].

### DNA damage response and tumour progression in squamous cell carcinogenesis

Given that keratinocytes are under constant exposure to UVR, the most important skin carcinogen, it is possible that the skin has adapted to this very situation. The presence of pATM in the cytoplasm, in addition to its DNA damage sensing role in the nucleus, may act to further protect the keratinocyte, perhaps via a cell signalling mechanism discussed above. With regard to expression patterns of pATM in tumours, it can be seen that early pre-invasive lesions - AK and CIS - express pATM to a significantly greater extent than SCCs. This is in keeping with the model proposed by Bartkova at al. that the DNA damage response acts as a barrier for tumour progression.

In contrast to the nuclear localisation of pATM in premalignant lesions (Figs 8 and 9) and in chronically UV exposed normal skin (Fig 5), pATM is cytoplasmic in normal skin from a non-UV exposed site (Fig 4), suggesting that chronic rather than acute UV exposure is required as a stimulus for nuclear pATM expression even though UV exposure in the short term leads to a transient increase in pATM levels as seen in Fig 4, albeit in the cytoplasm. The reduced nuclear expression of pATM in SCCs compared with precancerous lesions, suggests that other oncogenic stresses have overcome this response. One explanation is that mutations compromising the DNA damage response pathway, such as defects in pATM and its downstream effector molecules might allow cell proliferation and tumour progression.

There remain many unanswered questions to be addressed in future studies. pATM is consistently highly expressed in AKs and CIS, and it is unclear why some lesions progress and others do not. It is likely that that there are additional defects in cell cycle arrest, senescence and apoptosis which could be investigated such as cyclin E, Cdc25A and E2F1, to examine promotion of unscheduled S-phase entry [29], [30], as well as ATR, H2AX, phosphorylated forms of p53 and activated Chk1/2. In addition it is known that about one quarter of AKs regress [31] and this would be an interesting subset of lesions to investigate in this context. What is also not clear is whether early SCCs that apparently arise *de novo* without clinical or histological evidence of AK or CIS behave in a similar manner to AKs and CIS or whether they fail to mount a significant DNA damage response from the outset. Such studies may ultimately identify important diagnostic, prognostic and therapeutic biomarkers for improved skin cancer prevention and treatment.

## Materials and Methods

### Antibodies

We used primary antibodies against Ser 1981-phosphorylated ATM (Rockland), ATM (Abcam), Lamin A (Abcam), Actin (Abcam) and Giantin Golgi marker (Abcam). Secondary antibodies used for immunofluorescence included Alexa Fluor 568 and 488 (Molecular Probes). Secondary antibodies used for Western blotting were horseradish peroxidase-conjugated rabbit anti-mouse (Dako).

### Immunohistochemistry

Tumours were obtained from archival tissue banks in the histopathology department at Barts and The London NHS Trust. Ethics approval for the study of the molecular pathogenesis of non-melanoma skin cancers was gained by the Tissue Research Subcommitee of the East London and The City Health Authority Ethics. Written consent was obtained from all participants of this study. All sections used in this study were examined by Professor Rino Cerio (Professor of dermatopathology) and graded histologically. Control tissue was obtained directly from skin belonging to a 30 year old Caucasian female, from a non UV-exposed site, following abdominal reduction surgery. Normal UV exposed skin was obtained from an aged matched Caucasian female from the face. The skin was immediately prepared by removing connective tissue and subcutaneous fat, washed in warm sterile PBS and fixed with 4% PFA overnight. For UVB treated skin, sections were irradiated with 10 mJ/cm^2^ UVB. Pre-warmed E4+F12+RM+ media was then added (see below) and the tissue left at 37°C till the appropriate time for fixation post-UV, then immediately paraffin embedded for sectioning. Indirect immunoperoxidase staining on de-paraffinised tissue was performed using the Vectastain® Universal Elite ABC kit (Vector laboratories) and counterstained with haematoxylin. The immunostaining patterns were evaluated by two independent observers (FI and Dr Catherine Harwood) and the grade of protein expression scored as follows: 1, >0–5%; 2, >5–25%; 3, >25–50%; 4, >50–75%; 5, >75–100%

### Fluorescent and non-fluorescent immunocytochemistry

All monolayer cells were fixed with paraformaldehyde and permeabilised with 0.1% Triton X-100. 0.2% fish skin gelatine was used as a blocking agent followed by incubation with the primary antibody and secondary antibodies. Where more than one primary antibody was used in a single experiment, the relevant secondary antibody was added directly afterwards, then an additional blocking step included prior to incubation with the second set of primary and secondary antibodies. DAPI was used as a nuclear counter stain. Signal was visualised by fluorescent confocal microscopy (Carl Zeiss, Jena, Germany) and analysed using LSM 5 image examiner software. Cells non-fluorescently labelled were prepared for staining in the same way as for those fluorescently stained with regards to the permeabilisation, blocking and primary antibody steps described but this was then followed by incubation with Vectastain® Universal Elite ABC kit (Vector laboratories) and counterstained with haematoxylin.

### Cell culture

Keratinocyte-derived cell lines were grown in a mixture of 3 parts DMEM and 1 part Hams F12 medium with 10% FCS and the addition of various mitogens (RM+). 4 ml of RM+ concentrate was added to 440 ml of keratinocyte base medium to give final concentrations of Hydrocortisone 0.4 µg/ml, Cholera toxin 10^−10^ M, Transferrin5 µg/ml, Liothyronine 2×10^−11^ M, Adenine 1.8×10^−4^ M, Insulin 5 µg/ml, Epidermal Growth Factor 10 ng/m. Defined Keratinocyte serum-free media (Gibco®) was used for the growth of primary human keratinocyte cells. Cells were grown at 37°C in 5% CO_2_ UVB irradiation of cells was carried out using a CL-1000 ultraviolet cross linker (UVP) fitted with F8-T5 UV-B lamps with peak output at 312 nm, and a single dose of 10 mJ/cm^2^ used.

### Western Blotting

Cells were washed with PBS, collected by scraping and lysed in RIPA buffer (150 mM NaCl, 50 mM Tris pH 7.5, 1% Nonidet P-40, 0.05% sodium deoxycholate, 1% SDS and Roche protease inhibitor cocktail). Protein quantification was carried out using Bradford protein assay (Perbio Science UK). The resulting protein extracts were then separated by SDS-polyacrylamide gel electrophoresis and electroblotted onto nitrocellulose membranes. Reactive proteins were visualized by chemiluminescence with ECL plus (Amersham).

### Nuclear fractionation

All steps were performed on ice or at 4°C. Cells were collected and washed with ice-cold PBS then nuclei were isolated by Dounce homogenization in CaRSB buffer (10 mM NaCl, 1.5 mM CaCl2, 10 mM Tris-Cl, pH 7.5) and MS buffer (210 mM Mannitol, 70 mM sucrose, 5 mM EDTA, 5 mM Tris-Cl, pH 7.6). The homogenate was centrifuged at 800 g for 5 minutes. The nuclear pellet was washed with MS buffer, re-pelleted and resuspended in RIPA buffer with protease inhibitor cocktail.

### RNA interference

Cells were transfected with siRNA using HiPerFect (Qiagen) according to the manufacturer's instructions. Optimisation of the siRNA protocol was performed using siGLO Cyclophilin B positive control siRNA (Dharmacon). PM1 cells were silenced with ON-TARGETplus SMART pool human ATM siRNA or ON-TARGETplus non-targeting pool negative control siRNA (both from Dharmacon). Transfection complexes were removed after 24 hours and cells incubated in fresh medium for a further 48 hours before UV irradiation and harvesting for Western blot analysis or fixing for immunocytochemistry.

## Supporting Information

Figure S1 **Western blot of NHPK nuclear fraction +/− UV for pATM and ATM.** Nuclear extracts of NHPK were prepared with undamaged and UVB irradiated lysates examined for pATM and ATM expression using Lamin A as a nuclear loading control.
(TIF)

Figure S2 **Western blot of Scrambled and ATM silenced PM1 cells +/− UV with pATM antibody.** ATM in human keratinocytes (PM1) was silenced using SiRNA (Dharmacon). pATM antibody was directed against both undamaged and UVB irradiated scrambled and ATM silenced cells.
(TIF)

Figure S3 **Immunohistochemistry of Scr and ATM silenced PM1 cells +/− UV with pATM.** Both the scrambled and ATM silenced cells were plated onto glass coverslips, treated with UVB (10 mJ/cm2) and fixed at 1, 2 and 4 hours post-UVB. The cells were then labelled with pATM.
(TIF)
