# Supplementary figures and images for: Cutaneous Squamous Cell Carcinoma (SCC) and the DNA Damage Response: pATM Expression Patterns in Pre-Malignant and Malignant Keratinocyte Skin Lesions
Source: PLoS One. 2011 Jul 1;6(7):e21271. doi: 10.1371/journal.pone.0021271 (PMC3128585; doi:10.1371/journal.pone.0021271)

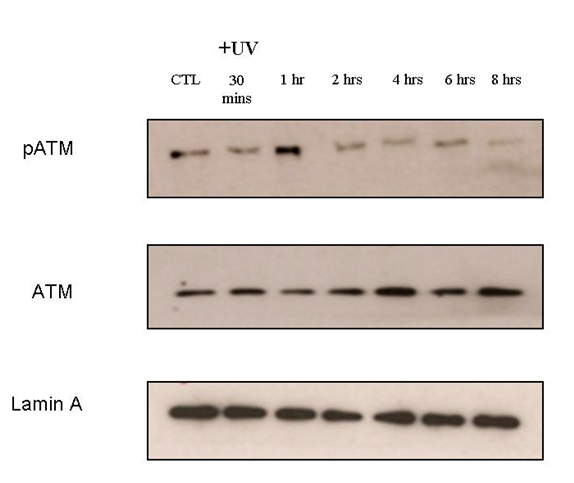

Supplement: Figure S1 — Western blot of NHPK nuclear fraction +/− UV for pATM and ATM. Nuclear extracts of NHPK were prepared with undamaged and UVB irradiated lysates examined for pATM and ATM expression using Lamin A as a nuclear loading control. (TIF) [file pone.0021271.s001.tif]

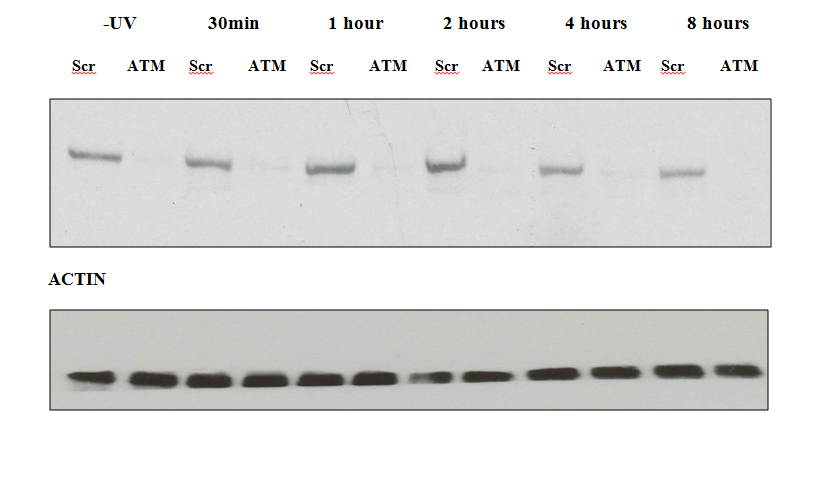

Supplement: Figure S2 — Western blot of Scrambled and ATM silenced PM1 cells +/− UV with pATM antibody. ATM in human keratinocytes (PM1) was silenced using SiRNA (Dharmacon). pATM antibody was directed against both undamaged and UVB irradiated scrambled and ATM silenced cells. (TIF) [file pone.0021271.s002.tif]

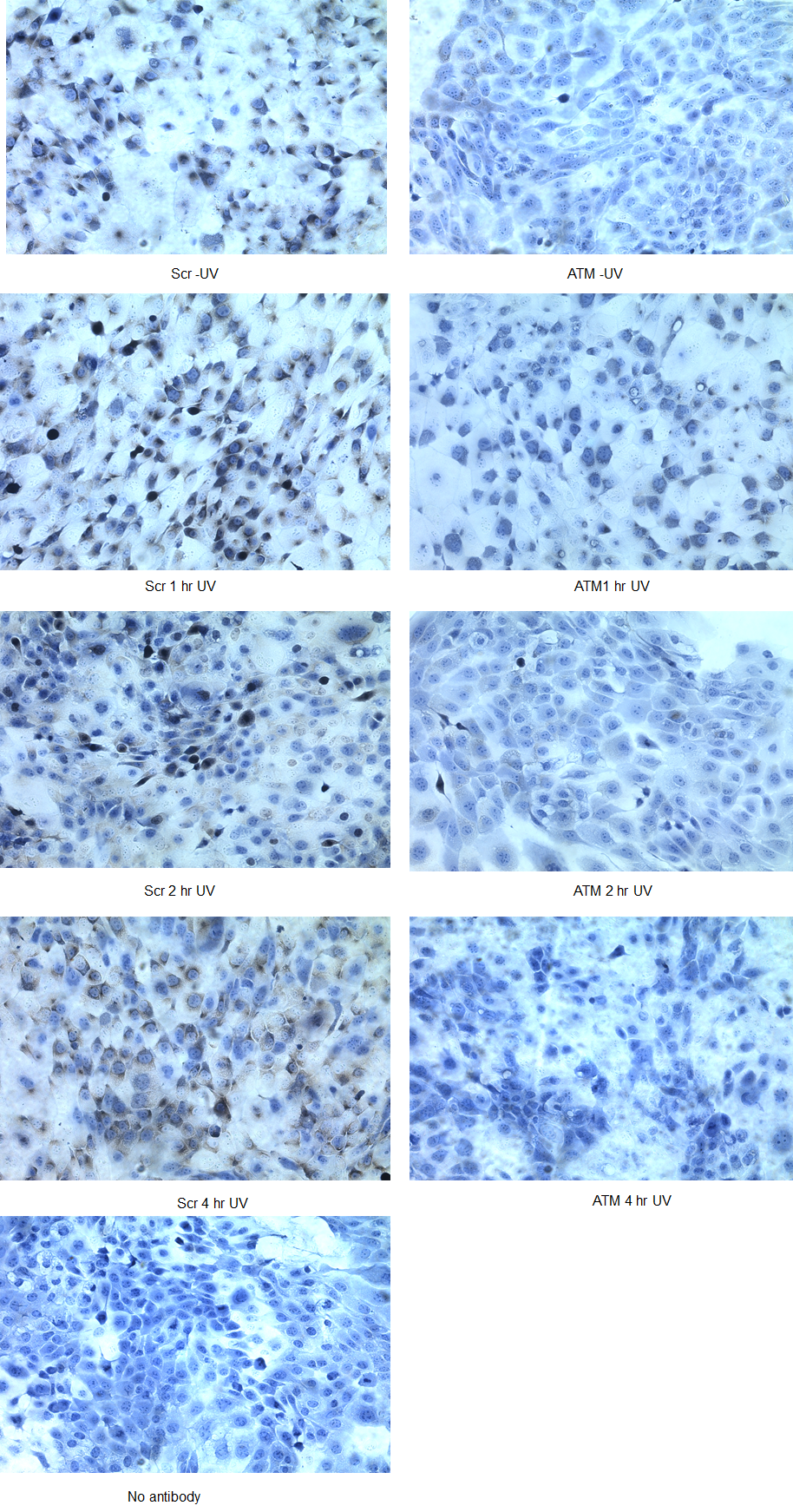

Supplement: Figure S3 — Immunohistochemistry of Scr and ATM silenced PM1 cells +/− UV with pATM. Both the scrambled and ATM silenced cells were plated onto glass coverslips, treated with UVB (10 mJ/cm2) and fixed at 1, 2 and 4 hours post-UVB. The cells were then labelled with pATM. (TIF) [file pone.0021271.s003.tif]
